# Supplementary material for: Modelling the effects of perceived system quality and personal innovativeness on the intention to use metaverse: a structural equation modelling approach
Source: PeerJ Comput Sci. 2024 Oct 8;10:e2331. doi: 10.7717/peerj-cs.2331 (PMC11623023; doi:10.7717/peerj-cs.2331)
Supplement: Supplemental Information 2 [file peerj-cs-10-2331-s002.docx]

**Metaverse Questionnaires**

**Gender:**

**Academic program:**

diploma

Bachelor's

Master's

**Personal Innovativeness**

If I heard about a metaverse, I would look for ways to experiment with it.

Among my peers, I am usually the first to try out metaverse.

I like to experiment with metaverse.

In general, I am hesitant to try out metaverse.

**System quality**

SQ1 The layout of the information in Metaverse is easy to

follow

SQ2 Metaverse allows me to find information easily

SQ3 It is easy for me to complete a transaction through metaverse

SQ4 I do not encounter long delays when searching for information in metaverse

SQ5 metaverse is visually appealing

SQ6 I feel secure in providing sensitive information through metaverse

**Perceived Usefulness**

PU1 The use of metaverse helps me to access learning resources

PU2 Using metaverse will improve learning performance in distance learning during

the COVID 19 pandemic

PU3 The use of metaverse will increase my productivity in distance learning during the

COVID 19 pandemic

PU4 The use of metaverse is beneficial for my learning activities

**Perceived Ease of Use**

PEU2 Learning to use metaverse is easy

PEU3 It is easy to navigate in metaverse

PEU4 The use of metaverse is flexible

**Behavior Intention**

BI1 I want all courses are offered via metaverse

BI2 I will use metaverse if it is available

BI3 I will recommend using metaverse in the future
