# Supplementary figures and images for: Modelling the effects of perceived system quality and personal innovativeness on the intention to use metaverse: a structural equation modelling approach
Source: PeerJ Comput Sci. 2024 Oct 8;10:e2331. doi: 10.7717/peerj-cs.2331 (PMC11623023; doi:10.7717/peerj-cs.2331)

Appendix 1

**
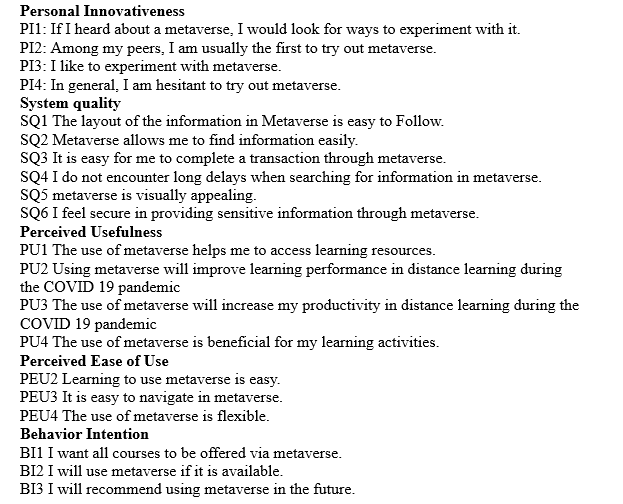
**

Supplement: Supplemental Information 3 [file peerj-cs-10-2331-s003.docx]
